# A Semantic SLAM System for Catadioptric Panoramic Cameras in Dynamic Environments

**DOI:** 10.3390/s21175889

**Published:** 2021-09-01

**Authors:** Yu Zhang, Xiping Xu, Ning Zhang, Yaowen Lv

**Affiliations:** School of Opto-Electronic Engineering, Changchun University of Science and Technology, Changchun 130022, China; zhangyucust@163.com (Y.Z.); zhangning@cust.edu.cn (N.Z.); lvyaowen2005@163.com (Y.L.)

**Keywords:** SLAM, semantic segmentation, multi-view geometry, dynamic environments

## Abstract

When a traditional visual SLAM system works in a dynamic environment, it will be disturbed by dynamic objects and perform poorly. In order to overcome the interference of dynamic objects, we propose a semantic SLAM system for catadioptric panoramic cameras in dynamic environments. A real-time instance segmentation network is used to detect potential moving targets in the panoramic image. In order to find the real dynamic targets, potential moving targets are verified according to the sphere’s epipolar constraints. Then, when extracting feature points, the dynamic objects in the panoramic image are masked. Only static feature points are used to estimate the pose of the panoramic camera, so as to improve the accuracy of pose estimation. In order to verify the performance of our system, experiments were conducted on public data sets. The experiments showed that in a highly dynamic environment, the accuracy of our system is significantly better than traditional algorithms. By calculating the RMSE of the absolute trajectory error, we found that our system performed up to 96.3% better than traditional SLAM. Our catadioptric panoramic camera semantic SLAM system has higher accuracy and robustness in complex dynamic environments.

## 1. Introduction

With the development of the mobile robot industry, robots increasingly require their own positioning when performing a variety of complex tasks. At present, visual simultaneous localization and mapping (SLAM) technology is one of the most common methods used to realize robot localization [1]. However, the current visual SLAM systems typically use a pinhole camera with a limited field of view, and assume that the scene is static. This assumption imposes many restrictions on the application scenarios of visual SLAM. In order to improve the accuracy and stability of the visual SLAM, it is an urgent task to use a panoramic camera with large field of view to improve the accuracy of the visual SLAM system in dynamic environments.

The distribution of features in the scene observed by the camera is one important factor that affects the performance of visual SLAM. As the camera field of view (FOV) increases, the number of features observed will also grow. When large field of view cameras are used in SLAM, it has more precise and robust performance than when ordinary pinhole cameras are used. A catadioptric panoramic camera can provide a 360-degree view of the scene, at a lower cost than a fisheye camera; thus, we chose to apply a catadioptric panoramic camera to the SLAM task.

In the past few decades, a variety of SLAM and visual odometry systems have been proposed. Some representative examples are ORB-SLAM2 [2], LSD-SLAM [3], and direct sparse odometry (DSO) [4]. ORB-SLAM2 is a visual SLAM algorithm that uses feature points to solve the camera pose and spatial points. LSD-SLAM is the first monocular Visual SLAM algorithm that uses the direct method to output semi-dense maps and optimized camera poses. DSO is a direct sparse odometry that joins the optimization of all parameters, including camera poses, camera intrinsics, and inverse depth value of space points.

There are also many studies on panoramic camera SLAM and visual odometry. Caruso et al. proposed a real-time, direct monocular SLAM method based on LSD-SLAM by incorporating the unified camera model. Heng et al. presented a semi-direct visual odometry for a fisheye-stereo camera [5]. In [6], a direct visual odometry for a fisheye-stereo camera was proposed. In [7] a unified spherical camera model was used for the fisheye camera, which was improved with the ORB-SLAM2 semi-dense map.

These methods are all based on the assumption that the objects in the scene are static. When the extracted feature points are from dynamic objects in the scene, it will affect the accuracy of the camera pose estimation. However, in reality there are not many completely static scenes; most scenes contain dynamic objects which will occlude the features being tracked, which may result in reduced accuracy of pose solution or loss of system tracking. Therefore, improving the accuracy and stability of SLAM in dynamic scenarios is an important research direction [8].

## 2. Related Work

The SLAM system in the classic dynamic environment does not consider using machine learning to detect dynamic targets. The dynamic objects in the environment are considered as outliers. The typical algorithms for removing outliers are RANSAC [9] and robust cost functions [10]. Sun et al. [11] proposed an online motion elimination method based on an RGB-D camera. They identified the moving foreground pixels based on the reprojection error, compared the current frame RGB-D image with the foreground model pixels, and moved the foreground. Pixels are divided to achieve the purpose of removing dynamic points. Li et al. [12] proposed a real-time depth edge-based RGB-D SLAM system for dynamic environments. It calculates the possibility that each key frame point is a static point, using this static weighting method to reduce the estimation of the camera pose by dynamic objects’ impact. Cheng, Sun, and Meng [13] used monocular cameras to distinguish dynamic points from static points through an optical-flow and a five-point algorithm approach to improve the accuracy of camera pose estimation.

Although these geometric-based visual SLAM systems can eliminate the influence of dynamic objects in the scene to a certain extent, they also have certain limitations. These methods lack semantic information and cannot use prior knowledge of the scene to judge the motion state of the object. Therefore, many SLAM systems combined with semantic segmentation networks have been proposed to deal with dynamic objects in complex environments.

Xiao et al. [14], based on convolutional neural networks, built an SSD object detector combined with prior knowledge, which they used to detect dynamic objects in the scene. The dynamic object detector was combined with the SLAM system. The selective tracking algorithm in the tracking thread was used to process the feature points of the dynamic objects, which significantly reduced the error of the camera pose estimation. However, the SSD object detector can only detect the boxes of dynamic objects in the image. In order to further improve the accuracy of the system, it is necessary to realize the detection of dynamic objects at the pixel level. Bescos et al. [15] used Mask R-CNN to detect dynamic targets, with the purpose of removing dynamic feature points. Nevertheless, their system is a dynamic SLAM system based on an RGB-D camera, and the camera cost is too high. In order to realize the SLAM system with low cost and high precision, it is very important to select a low-cost monocular catadioptric panoramic camera as the sensor in the SLAM system. Yu et al. [16] employed SegNet for the detection of moving objects, which improved the accuracy and stability of the SLAM system. However, since they used a pinhole camera with a limited field of view, when there are many dynamic objects in the image, after removing the dynamic objects, the available features in the image may be insufficient. In such instances, the camera pose calculation fails.

In order to solve the above problems, we propose a catadioptric panoramic camera semantic SLAM system for dynamic environments.

The rest of the paper is structured as follows: In Section 2, a brief introduction to semantic segmentation of potentially dynamic features is given. We then analyze the spherical surface pole constraints according to the projection model of the catadioptric panoramic camera, and, combining the results of semantic segmentation of panoramic images, a method is proposed to eliminate dynamic points in a tightly coupled manner between the semantic results and the sphere epipolar geometry. In Section 3 we evaluate the accuracy of our system on the Omni dataset and compare our system with state-of-the-art SLAM systems. Finally, a summary is provided in Section 4.

## 3. Methods

### 3.1. Panoramic Image Semantic Segmentation

Knowing the types of objects in the scene can help us deal with complex tasks in a dynamic environment. However, traditional methods cannot provide this prior information. With the rapid development of deep learning, semantic segmentation networks can output pixel-level semantic information. Therefore, combining semantic segmentation networks with traditional SLAM systems can complete complex tasks in dynamic environments. Because the semantic information is used to mark the potential dynamic points in the environment, the image is input into the SLAM system with prior information.

MASK-RCNN is a representative instance segmentation network [17], but it cannot meet the real-time requirements in SLAM. In order to be able to input images with semantic information for the SLAM system in real time, we use YOLACT [18] to perform instance segmentation on panoramic images. Through training, it can detect potential moving objects including cars and people.

YOLACT divides instance segmentation into two parallel tasks: Generating a non-local prototype mask over the entire image and predicting a set of linear combination coefficients per instance. It then generates a full-image instance segmentation from these two components: For each instance, the prototype is linearly combined with the corresponding prediction coefficient, then the predicted bounding box is used for cropping [8].

**Figure 1:**
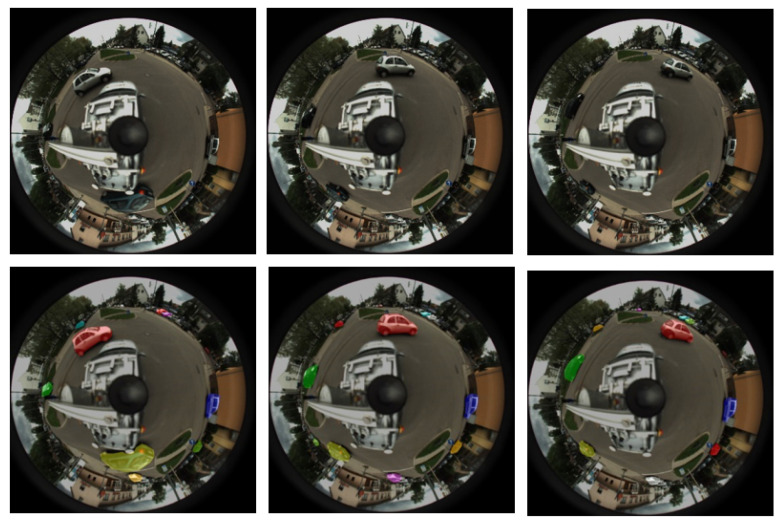
Semantic segmentation results.

The size of the original RGB image input to the network structure is h×w×3, the output of the network is a matrix of size h×w×n, and *n* represents the number of instances of cars and people in the image. Figure 1 shows the image we input to the YOLACT network and the result of instance segmentation.

### 3.2. Dynamic Point Removal

Through the results of instance segmentation output by the YOLACT instance segmentation network, we obtain pixel-level semantic information of the panoramic image. Under normal circumstances, according to the semantic label corresponding to the pixel in the panoramic image, the type information in each instance can be distinguished, the corresponding dynamic instance can be eliminated, and the pose estimation of the catadioptric panoramic camera is not involved. However, vehicles in reality are not all dynamic. When a static vehicle occupies most of the view of the panoramic image, if the vehicle is removed as a dynamic point at this time, a large amount of useful information will be lost, or feature points will be insufficient and the camera pose calculation will fail.

The result of YOLACT instance segmentation has nothing to do with the motion state of the objects in the scene. Semantic tags cannot be used to distinguish the motion state of objects in the scene. We need a method to distinguish the motion state of objects and reflect the real movement of objects in the scene.

After analyzing the projection model of the catadioptric panoramic camera [19], the sphere epipolar constraint is used to verify whether the feature points in the panoramic image are dynamic or static. If a point is static, for a pair of matching feature points in two panoramic images, the feature vector obtained by back projection should intersect the polar line on the sphere., and the dynamic feature point does not satisfy this constraint.

**Figure 2:**
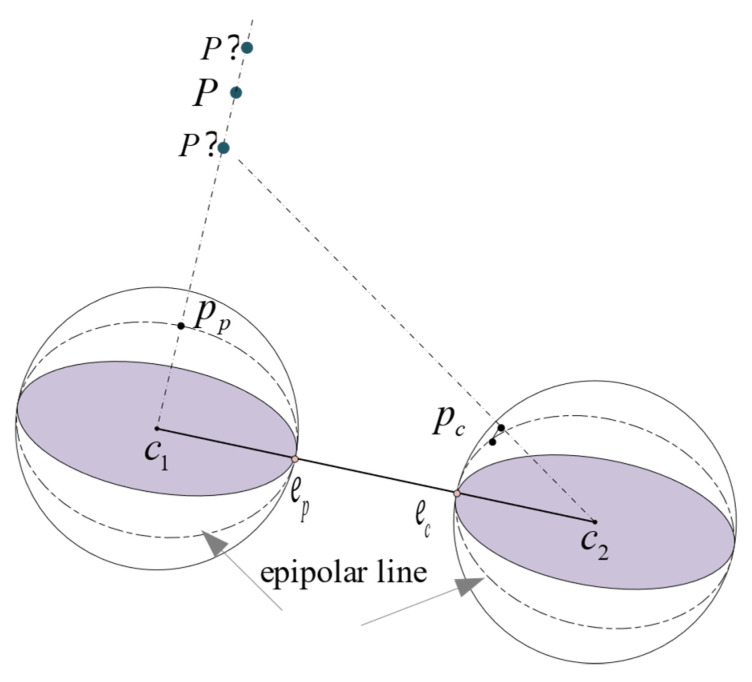
Sphere epipolar geometry.

In Figure 2, point P is a spatial point observed by both the previous and current frames. $p_{p}=\left(\begin{array}{ccc} u_{sp} & v_{sp} & w_{sp} \end{array}\right)$ and $p_{c}=\left(\begin{array}{ccc} u_{sc} & v_{sc} & w_{sc} \end{array}\right)$, respectively, correspond to the unit vectors of the two frames of panoramic images that are back-projected from the catadioptric panoramic camera projection model to the unit sphere, and $\sqrt{u_{s}{}^{2}+v_{s}{}^{2}+w_{s}{}^{2}}=1$. The line $c_{1}c_{2}$ connecting the center points of the two spheres is the baseline. The intersection points $e_{p}$ and $e_{c}$ of the baseline and the two spheres are called epipoles. Suppose we know $p_{p}$ in the left sphere and want to find the corresponding $p_{c}$ in the right sphere. Without depth information, we know that the space point *P* is on the corresponding ray $\vec{c_{1}p_{p}}$, but the specific position on the line is not known. When the spatial point *P* is static, there should be a correct feature match, and we can know that point $p_{c}$ is on the epipolar line of the sphere on the right. This geometric constraint is the sphere epipolar constraint of the catadioptric panoramic camera.

(1) $$p_{p}{}^{T}t^{\wedge }Rp_{c}=0$$

(2) $$E=t^{\wedge }R$$

(3) $$t^{\wedge }=\left[\begin{array}{ccc} 0 & -t_{3} & t_{2}\\ t_{3} & 0 & -t_{1}\\ -t_{2} & t_{1} & 0 \end{array}\right]$$

***R*** is the rotation matrix, ***t*** is the translation vector and E is the essential matrix. According to the epipolar constraint, when the point *P* is a static point, $p_{p}$ and $p_{c}$ must satisfy the constraint. However, due to the error of feature matching and the uncertainty of the essential matrix, in the actual solution process the static point may not strictly meet the constraint, when $p_{c}$ is not exactly on the line but some distance away from the epipolar line. In such a case we can set a threshold; when the distance is less than the threshold, the point is considered to be a static point, and when the distance is greater than the threshold, it is a dynamic point.

We need to request the essential matrix before we can use the sphere epipolar constraint to determine the state of the scene point. Usually, E has five degrees of freedom, indicating that we can use at least five pairs of matching feature points to solve E, but the inherent property of E is a non-linear property, which is troublesome to estimate, and thus we use the eight-point-algorithm to solve E. When we have a pair of matching points whose back-projection vectors are $p_{p}=\left(\begin{array}{ccc} u_{sp} & v_{sp} & w_{sp} \end{array}\right)$ and $p_{c}=\left(\begin{array}{ccc} u_{sc} & v_{sc} & w_{sc} \end{array}\right)$, according to the epipolar constraint:

(4) $$\left(\begin{array}{ccc} u_{s2} & v_{s2} & w_{s2} \end{array}\right)\left(\begin{array}{ccc} e_{1} & e_{2} & e_{3}\\ e_{4} & e_{5} & e_{6}\\ e_{7} & e_{8} & e_{9} \end{array}\right)\left(\begin{array}{c} u_{s1}\\ v_{s1}\\ w_{s1} \end{array}\right)=0$$

The E expansion is written in vector form:

(5) $$e={\left[e_{1},e_{2},e_{3},e_{4},e_{5},e_{6},e_{7},e_{8},e_{9}\right]}^{T}$$

Formula (4) can be written as a linear formula related to e:

(6) $$\left[u_{s2}u_{s1},u_{s2}v_{s1},u_{s2},v_{s2}u_{s1},v_{s2}v_{s1},v_{s2},u_{s1},v_{s1},1\right]\cdot e=0$$

There are nine unknowns in Formula (6), but due to the equivalence of scale of E, the degrees of freedom of e can be reduced to eight. At this time, we select eight pairs of matching feature points between consecutive image frames and solve Equation (6) to obtain E.

**Figure 3:**
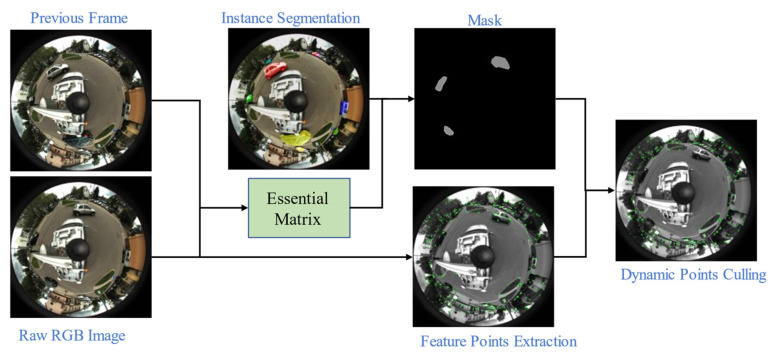
Overall framework of dynamic point elimination.

With the essential matrix, E, and the result of instance segmentation, a thread of dynamic point elimination can be added to the SLAM system. Figure 3 presents the overall algorithm framework we propose to eliminate dynamic points in the catadioptric panoramic image. First, the essential matrix between the previous frame and current frame of the catadioptric panoramic camera model is calculated, and the instance segmentation network is used to find the potential dynamic objects. Then, the sphere epipolar constrained is used to further verify the pixels of the potential dynamic objects, the real dynamic objects are found, and a mask image is generated; finally, the dynamic points in the panoramic image are eliminated according to the obtained mask image during the detection of the ORB feature points.

## 4. Experiment and Analysis

### 4.1. Public Dataset Experiment

**Figure 4:**
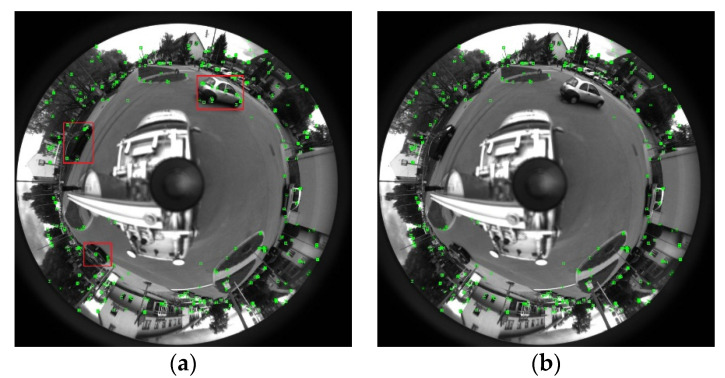
Dynamic point elimination results (**a**) Raw ORB feature points extract method and (**b**) ORB feature points extracted by our Method.

In order to verify the performance of the SLAM system based on the catadioptric panoramic camera in a dynamic environment, experiments were conducted on the Omnidirectional public dataset [20]. This dataset was collected by a car loaded with two catadioptric panoramic cameras on a city street, and contains 12,607 frames of images in total. There are a large number of dynamic and static cars and people in the images, which is very suitable for testing the performance of the SLAM system of the catadioptric panoramic camera in a dynamic environment. Figure 4 shows the result of feature point extraction in the original image and after removing the dynamic objects. After implementing our dynamic object culling method, the moving cars selected in the box in (a) were all successfully eliminated when extracting feature points in (b).

A number of image sequences containing dynamic objects were selected in the dataset, and we divided them into low dynamic sequences, high dynamic sequences and sequences containing high dynamic and low dynamic objects. To verify the SLAM algorithm proposed in this paper, we evaluated the absolute trajectory error (ATE) and relative pose error (RPE) indicators for our SLAM system. ATE is used to calculate the error between the estimated trajectory and the ground truth, which can directly reflect the accuracy and global consistency of the pose estimation algorithm, and is usually used to evaluate the performance of the entire SLAM system. RPE is an indicator for evaluating system drift. It calculates the difference between two pose changes in a fixed period of time.

(7) $$\mathrm{ATE}=\sqrt{\frac{1}{N}{\sum_{i=1}^{N}\left\|\log{\left(T_{gt,i}{}^{-1}T_{esti,i}\right)}^{\vee }\right\|}_{2}^{2}}$$

Formula (7) is used to calculate RMSE of ATE. $T_{esti,i}$ represents the trajectory of the calculated Lie algebra form. $T_{gt,i}$ represents the trajectory of the Lie algebraic form of ground truth, for i=1,⋯,N. *N* represents the total number of poses included in the trajectory.

**Figure 5:**
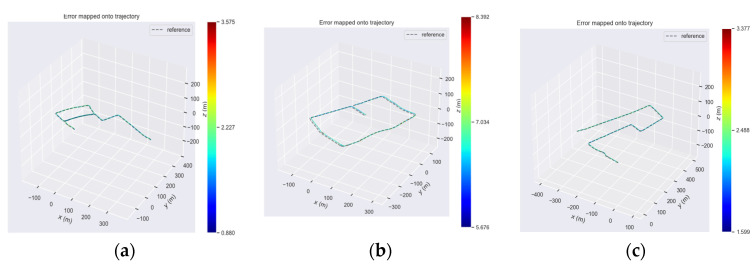
Average relative translation error between our method and groundtruth: (**a**) result of low dynamic sequence, (**b**) result of high dynamic sequence, (**c**) result of sequences containing high dynamic and low dynamic objects.

We used RMSE, mean error, median error, and standard deviation (SD) to evaluate ATE indicators, and average relative translation to evaluate RPE indicators. The trajectory calculated using our method was compared with the monocular catadioptric panorama VO method and the ground truth. The monocular catadioptric panoramic VO [21] is based on the ORB-SLAM2 improved VO algorithm that is suitable for catadioptric panoramic cameras. We collected statistics on the calculated data including the RMSE, mean error, median error, and standard deviation, and recorded them in Table 1. We then used average relative translation to evaluate the RPE index. Figure 5 shows the error between our calculated trajectory and the ground truth. The relative error can be seen by the color. Analysis of the figure shows that the error between our calculated trajectory and the ground truth remained within a small range of changes and there was no sudden change and drift of the trajectory due to dynamic objects, which proves the robustness of the algorithm presented this paper.

**Figure 6:**
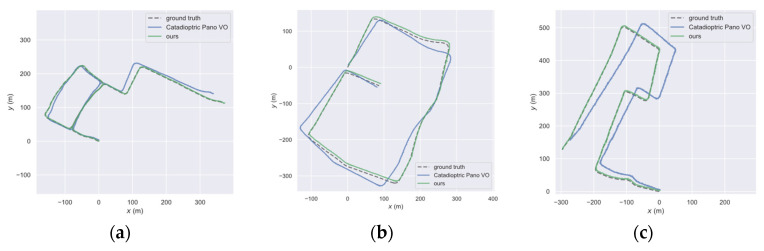
Comparison of absolute trajectory error (ATE) on public dataset (**a**) result of low dynamic sequence (**b**) result of high dynamic sequence (**c**) result of sequences containing high dynamic and low dynamic objects.

Figure 6 shows the result of comparison of the trajectories obtained by different methods and the groundtruth. The trajectory calculated by our system was closer to the groundtruth, while the improved Catadioptric Pano VO based on ORB-SLAM2 was the closest to our calculated trajectory in a low dynamic environment, because ORB-SLAM2 uses the RANSAC algorithm in a low dynamic environment. As outliers, dynamic points are not used for pose estimation, which is less affected by dynamic points. There were highly dynamic targets in the other two sequences, where the RANSAC algorithm failed, whereas our system could still effectively remove dynamic points. The trajectory accuracy calculated by our system was higher than the trajectory accuracy calculated by Catadioptric Pano VO and closer to the groundtruth. Analyzing the data in Table 1, we conclude that our system performed better than Catadioptric Pano VO in the three sequences: the low dynamic sequence, the high dynamic sequence, and the low and high dynamic sequence. By calculating the RMSE of absolute trajectory error, we compared our system with Catadioptric Pano VO, and found that our system improved the results by 20%, 95.2%, and 96.3% respectively. When facing complex dynamic environments, our system effectively removed dynamic points for pose estimation, and the accuracy of pose estimation was also greatly improved compared to traditional methods.

### 4.2. Real-World Experiment

**Figure 7:**
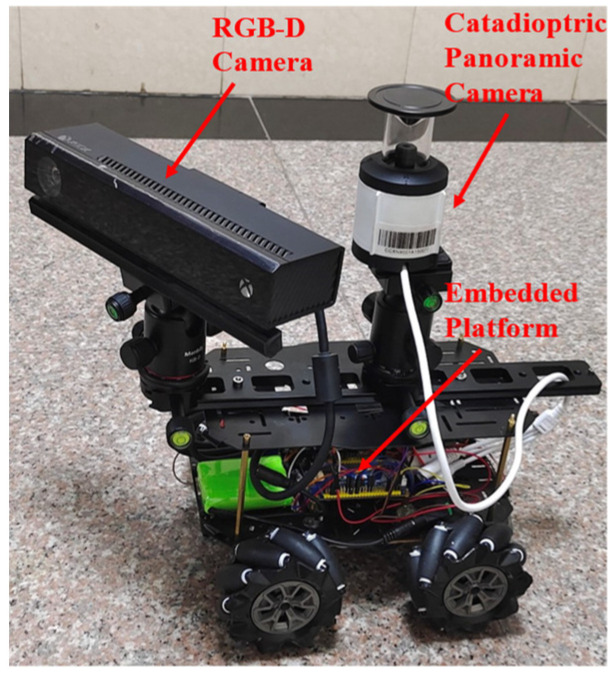
Experimental platform.

An indoor scene experiment on experimental platforms was conducted. As shown in Figure 7, our experimental platform was an unmanned ground vehicle (UGV) with an RGB-D camera and a catadioptric panoramic camera. An embedded development board in the platform was used to collect and store images. During the experiment, the maximum speed of our UGV did not exceed 3 m/s.

**Figure 8:**
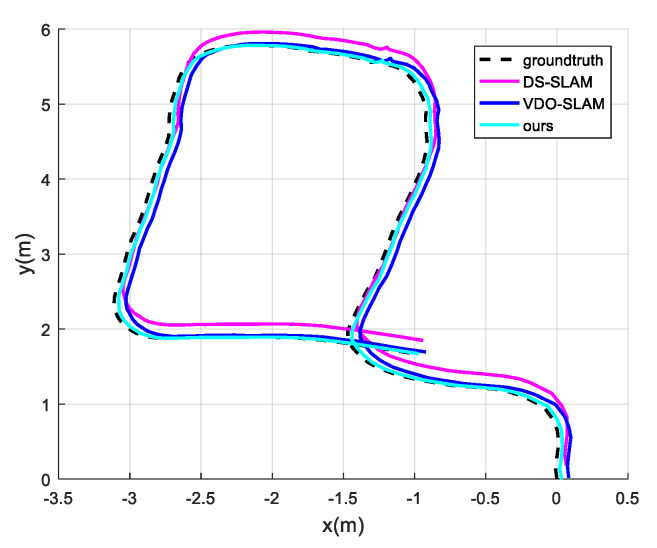
Comparison of experiment trajectories.

We set a fixed route, and the trajectory calculated from the images collected by the RGB-D camera in a static environment was used as the ground truth. During the experiment, we added walking humans as dynamic objects. Image data were collected by the RGB-D camera and the catadioptric panoramic camera. The collected image data were used to compare the performance of our method with the state-of-the-art dynamic SLAM system. DS-SLAM and VDO-SLAM [22] were adopted for comparisons. The trajectory obtained by different methods in the real-world experiment is shown in Figure 8. We used ATE to evaluate the accuracy of each method. The results are shown in Table 2.

In the experiments, DS-SLAM, VDO-SLAM and our method all ran stably in the environment with dynamic objects. Our method obtained the highest accuracy, with an improvement of 30.53% over DS-SLAM and 24.43% over VDO-SLAM. This is because we used a catadioptric panoramic camera, which has the advantage of a large field of view. After removing dynamic objects, there still are rich scene features that can be used to calculate the camera pose.

## 5. Conclusions

In this paper, we have presented a semantic visual SLAM system for a catadioptric panoramic camera in a dynamic environment building on ORB-SLAM2. We added a separate thread running YOLACT to obtain pixel-wise semantic segmentation, and a tight coupling method of spherical epipolar geometry and semantic segmentation was proposed to detect and remove dynamic features effectively. We used an instance segmentation network to detect potential moving targets in the image, output the pixel-level segmentation results, and then use sphere epipolar geometry to verify the motion state and find the real dynamic features. When extracting ORB feature points, dynamic targets are eliminated, thereby improving the accuracy and robustness of camera pose estimation. In order to verify the performance of our system, experiments were conducted on a public omnidirectional dataset. The results show that in a dynamic environment, the positioning accuracy of our system is greatly improved compared to the traditional method. Comparing the RMSE of absolute trajectory error of different methods, the positioning accuracy of our system in a high dynamic environment was up to 96.3% higher than the traditional method, which proves the effectiveness of our system when faced with dynamic environments.

We built an experimental platform and conducted real world experiments. We compared our method with the dynamic state-of-the-art SLAM system. As we use the semantic segmentation result and geometry to detect and remove dynamic objects in the image in a tightly coupled manner, and the catadioptric panoramic camera we use has the advantage of a large field of view, in the experimental results, our method obtained the highest accuracy, with improvements of 30.53% over DS-SLAM and 24.43% over VDO-SLAM.

However, our current system only uses two frames of information to verify the dynamic points, and lacks the use of global information. The next step should be to improve this aspect.

## Figures and Tables

**Table 1:** Comparison of absolute trajectory error (ATE) on the Omnidirectional public dataset.

Sequence	Catadioptric Pano VO				Ours			
	RMSE (× 10 m)	Median (× 10 m)	Mean (× 10 m)	S.D (× 10 m)	RMSE (× 10 m)	Median (× 10 m)	Mean (× 10 m)	SD (× 10 m)
Low dynamic sequence	0.012	0.011	0.010	0.003	0.0096	0.0095	0.0092	0.0029
Low and high dynamic sequence	0.972	0.971	0.972	0.218	0.0359	0.0355	0.0354	0.011
High dynamic sequence	0.923	0.914	0.916	0.304	0.0445	0.0440	0.0441	0.013

**Table 2:** Results of metrics of absolute trajectory error.

Method	RMSE (m)	Median (m)	Mean (m)
DS-SLAM	0.285	0.216	0.252
VDO-SLAM	0.262	0.203	0.237
Ours	0.198	0.131	0.091

## Data Availability

Not applicable.

## References

[1] Bresson G., Alsayed Z., Yu L., Glaser S. (2017). Simultaneous Localization and Mapping: A Survey of Current Trends in Autonomous Driving. IEEE Trans. Intell. Veh..

[2] Mur-Artal R., Tardós J.D. (2017). ORB-SLAM2: An Open-Source SLAM System for Monocular, Stereo, and RGB-D Cameras. IEEE Trans. Robot..

[3] Engel J., Schöps T., Cremers D. LSD-SLAM: Large-Scale Direct Monocular SLAM. Proceedings of the Computer Vision—ECCV 2014.

[4] Engel J., Koltun V., Cremers D. (2018). Direct Sparse Odometry. IEEE Trans. Pattern Anal. Mach. Intell..

[5] Heng L., Choi B. Semi-direct visual odometry for a fisheye-stereo camera. Proceedings of the 2016 IEEE/RSJ International Conference on Intelligent Robots and Systems (IROS).

[6] Liu P., Heng L., Sattler T., Geiger A., Pollefeys M. Direct visual odometry for a fisheye-stereo camera. Proceedings of the 2017 IEEE/RSJ International Conference on Intelligent Robots and Systems (IROS).

[7] Wang S., Yue J., Dong Y., Shen R., Zhang X. Real-time Omnidirectional Visual SLAM with Semi-Dense Mapping. Proceedings of the 2018 IEEE Intelligent Vehicles Symposium (IV), Real-time Omnidirectional Visual SLAM with Semi-Dense Mapping.

[8] Li G., Liao X., Huang H., Song S., Liu B., Zeng Y. (2021). Robust Stereo Visual SLAM for Dynamic Environments With Moving Object. IEEE Access.

[9] Chum O., Matas J., Kittler J. (2003). Locally Optimized RANSAC.

[10] Pire T., Fischer T., Civera J., Cristóforis P.D., Berlles J.J. Stereo parallel tracking and mapping for robot localization. Proceedings of the 2015 IEEE/RSJ International Conference on Intelligent Robots and Systems (IROS).

[11] Sun Y., Liu M., Meng M.Q.H. (2017). Improving RGB-D SLAM in dynamic environments: A motion removal approach. Robot. Auton. Syst..

[12] Li S., Lee D. (2017). RGB-D SLAM in Dynamic Environments Using Static Point Weighting. IEEE Robot. Autom. Lett..

[13] Cheng J., Sun Y., Meng M.Q.H. (2019). Improving monocular visual SLAM in dynamic environments: An optical-flow-based approach. Adv. Robot..

[14] Xiao L., Wang J., Qiu X., Rong Z., Zou X. (2019). Dynamic-SLAM: Semantic monocular visual localization and mapping based on deep learning in dynamic environment. Robot. Auton. Syst..

[15] Bescos B., Campos C., Tardós J.D., Neira J. (2021). DynaSLAM II: Tightly-Coupled Multi-Object Tracking and SLAM. IEEE Robot. Autom. Lett..

[16] Yu C., Liu Z., Liu X., Xie F., Yang Y., Wei Q., Fei Q. DS-SLAM: A Semantic Visual SLAM towards Dynamic Environments. Proceedings of the 2018 IEEE/RSJ International Conference on Intelligent Robots and Systems (IROS).

[17] He K., Gkioxari G., Dollár P., Girshick R. Mask R-CNN. Proceedings of the 2017 IEEE International Conference on Computer Vision (ICCV).

[18] Bolya D., Zhou C., Xiao F., Lee Y.J. YOLACT: Real-time Instance Segmentation. Proceedings of the 2019 IEEE/CVF International Conference on Computer Vision (ICCV).

[19] Scaramuzza D., Martinelli A., Siegwart R. A Flexible Technique for Accurate Omnidirectional Camera Calibration and Structure from Motion. Proceedings of the IEEE International Conference on Computer Vision Systems.

[20] Schönbein M., Geiger A. Omnidirectional 3D Reconstruction in Augmented Manhattan Worlds. Proceedings of the 2014 IEEE/RSJ International Conference on Intelligent Robots and Systems.

[21] Zhang Y., Xu X., Zhang N., Lv Y., Lu Y. (2021). Research on Visual Odometry Based on Catadioptric Panoramic Camera. Acta Photonica Sin..

[22] Zhang J., Henein M., Mahony R., Ila V. (2020). VDO-SLAM: A Visual Dynamic Object-aware SLAM System. arXiv.

